# Psychometric properties and factor structure of the diabetes eatıng problem survey- revised (DEPS-R) among adults with type 1 diabetes mellitus

**DOI:** 10.1007/s40519-023-01602-y

**Published:** 2023-09-04

**Authors:** Yasemin Atik-Altınok, Beyza Eliuz-Tipici, Cemile İdiz, Su Özgür, Ayşe Merve Ok, Kubilay Karşıdağ

**Affiliations:** 1 Division of Pediatric Endocrinology, Department of Pediatrics, Faculty of Medicine, Ege University, 35100 Bornova, İzmir Turkey; 2 Division of Pediatric Endocrinology, Department of Pediatrics, Istanbul University, Istanbul Faculty of Medicine, Istanbul, Turkey; 3 Division of Endocrinology and Metabolism, Department of Internal Medicine, Istanbul University, Istanbul Faculty of Medicine, Istanbul, Turkey; 4 Department of Biostatistics and Medical Informatics, Faculty of Medicine, Ege University, İzmir, Turkey

**Keywords:** DEPS-R, Distributed eating behaviors, Type 1 diabetes mellitus, Adults

## Abstract

**Background and objective:**

Although many studies on the Diabetes Eating Problem Survey–Revised (DEPS-R) in adolescents with type 1 diabetes mellitus (T1D), the number of studies validating this questionnaire in adults with T1D is limited. Therefore, this study aimed to examine the factor structure of the Turkish version of the DEPS-R in adults with T1D and internal consistency and construct validity.

**Methods:**

A total of 100 patients with T1D, ages 18–50 years, completed the DEPS-R and EDE-Q. In addition to tests of validity, confirmatory factor analysis was conducted to investigate the factor structure of the 6-item Turkish version of DEPS-R.

**Results:**

The Cronbach’s alpha coefficient of the DEPS-R Turkish version was 0.77, suggesting good internal consistency. The median (IQ) DEPS-R score was 15.0 (13.0) among all participants. DEPS-R score was significantly correlated with BMI (*r* = 0.210; p < 0.05) and EDE-Q (*r* = 0.586; p < 0.01). There was no correlation between the HbA1c values of participants and neither EDE-Q nor DEPS-R scores. The confirmatory factor analysis results show that the three-factor model was a good fit.

**Conclusion:**

A short, self-administered diabetes-specific screening tool for disordered eating behavior is recommended be used routinely in the clinical care of adults with T1D, and Turkish version of DEPS-R has acceptable internal consistency and construct validity in adults with T1D.

**Level of evidence:**

Level V, descriptive study.

*Clinicaltrials.gov registration number* NCT05346679/ 21.04.2022 (retrospectively registered)

## Introduction

The term ‘disordered eating behaviors (DEB)’ encompasses extreme dieting behavior, binge eating attacks, and compensatory behavior for weight control [1]. The etiology of DEB is complex and multifactorial. Individual, familial, and sociocultural factors can contribute to the development of DEB [2, 3]. The frequency of these disorders in girls and women with T1D is 2–3 times higher than in the general population [4–6]. Although most of the studies focus on young girls and females, adolescent males with T1D also may have an increased risk of development of DEB [7].

Many mechanisms have been proposed to explain the relationship between DEB and T1D. Diabetes management may be an iatrogenic factor, requiring increased attention to maintaining healthy weight control, focusing on food intake and glycemia, risk of insulin-related weight gain, and associated body dissatisfaction. This can eventually induce overeating and binge eating episodes and may then intensive efforts to control food intake and weight. For example, an individual with T1D may enter a vicious cycle of dieting, further binge eating, and weight control behavior [6, 8]. Insulin omission to reduce the body weight is a unique, type 1 diabetes-specific eating purging behavior. It promotes weight loss through hyperglycemia, osmotic diuresis, glycosuria, ketonuria, dehydration and by allowing to excretion of the energy obtained from food through urine [6]. Insulin underdosing or skipping insulin is associated with recurrent episodes of diabetic ketoacidosis, severe hyperglycemia episodes, poor metabolic control, and the development of macro and micro complications, dramatically increasing morbidity and mortality [9, 10].

Early detection and treatment of eating disorders in individuals with T1D are essential because of potentially severe consequences such as diabetic ketoacidosis, hospitalization, and diabetes-related medical complications, particularly retinopathy and neuropathy [6, 11]. Several screening questionnaires and structured clinical interviews help to identify and diagnose eating disorders in adolescents and adults; however, it is essential to use a screening questionnaire measure designed specifically for individuals with T1D for two reasons: (i) the standard questionnaire may overestimate the prevalence of DEB, because due diligence in food/carbohydrate intake for the management of T1D may be misinterpreted as an eating disorder; (ii) standard questions may underestimate the prevalence of DEB because they will overlook patients who deliberately omit insulin as a way to control/lose weight.

The Diabetes Eating Problem Survey–Revised (DEPS-R) is the first screening tool for disordered eating designed specifically for individuals with T1D. Its psychometric properties among individuals with T1D have been established in English, German, Turkish, Norwegian, Italian, and Chinese. In these validation studies, the prevalence of the risk of distributed eating behaviors detected with DEPS-R was between 15 and 39% [11–16]. Our previous study found that DEPS-R-positive cases had an 8.5-fold higher risk for eating disorders (ED) than DEPS-R-negative ones. In addition, these had a higher level of depression and anxiety than DEPS-R-negative ones. Moreover, DEPS-R-positive cases had greater emotion dysregulation. Separately investigating the subscales of DEPS-R, we found that these patients were more incapable of accessing emotion regulation strategies and engaging in goal-directed behavior while under challenging emotions and impulse control. Furthermore, the DEPS-R score was significantly positively correlated with anxiety, depression, emotion dysregulation levels, and the severity of attention deficit and hyperactivity disorder [17].

Furthermore, in recent studies, a confirmatory factor analysis supported previously reported three-factor solutions describing ‘maladaptive eating’, ‘preoccupation with thinness’, and ‘maintaining high blood glucose levels to lose weight’ in adolescents and adults [14, 18, 19]. The DEPS-R appears to be the best-validated tool for adolescents and adults with T1D [8]. This study aimed to examine the internal consistency and construct validity of the DEPS-R Turkish version in adults with T1D.

## Materials and methods

### Study design and participants

Patients with T1D were recruited from Istanbul University, Istanbul Medical Faculty Division of Endocrinology and Metabolism, diabetes outpatient clinic from March to October 2021. One hundred eighty four patients with T1D invited the study. This cross-sectional study was conducted with 163 participants with T1D aged 18–50 years. All participants answered a DEPS-R and EDE-Q questionnaire during a regularly scheduled medical visit. Written informed consent was obtained from all participants. Patient records were reviewed for the following eligibility criteria: duration of T1D ≥ 1 year, regular follow-up of at least 1 year, and no major medical problems (celiac disease, cystic fibrosis, psychiatric disorders, and communication difficulties). Finally, the data of 100 participants who meet the eligibility criteria and responded to all items of the DEPS-R and EDE-Q scales were included in the analysis. The sample size was in line with the measurement development literature recommending a minimum of 5–10 participants per questionnaire item for DEPS-R [20].

### Anthropometric evaluation

Height was measured using a stadiometer to the nearest 0.1 cm in all participants. Weight was measured unclothed to the nearest 0.1 kg using a calibrated balance scale. Body Mass Index (BMI) was calculated by using the weight (kg)/height (m^2^) equation. The participants were categorized into three groups according to BMI underweight (BMI < 18.5), normal weight (BMI 18.5–24.9), overweight (BMI 25.0–29.9), and obese (BMI > 30).

### Glycemic control

HbA1c measurements were performed with ion-exchange high-performance liquid chromatography (Bio-Rad Variant II Turbo, Japan).

### Screening distributed eating behaviors

Diabetes Eating Problem Survey-Revised (DEPS-R) is a 16-item diabetes-specific self-report questionnaire to test for the risk of diabetes-specific eating disorders. Answers are scored on a six-point Likert scale, with higher scores indicating more DEB and a total score of ≥ 20 indicating an increased risk for eating disorders (range 0–80). The DEPS-R has a three-factor model: incompatible eating habits, weight loss or preoccupation with body weight, and an approach to maintaining high blood glucose values to lose weight. The original DEPS-R has been shown to have a good internal consistency (Cronbach’s alpha = 0.86) and construct validity in a sample of the pediatric population with T1D [11]. The DEPS-R has been translated to Turkish and validated in adolescents, demonstrating good internal consistency (Cronbach’s alpha = 0.85) [13].

The Eating Disorder Examination Questionnaire (EDE-Q) is a 28-item self-report questionnaire on specific eating disorder psychopathology. It consists of four subscales eating restraint, eating concern, shape concern, and weight concern. The global score represents an average of the four subscale scores, with higher scores indicating greater eating pathology. The measure also includes six free-response items assessing the frequency of ED behaviors (e.g., binging, purging) [21]. The EDE-Q has been translated to Turkish and validated, demonstrating good internal consistency (Cronbach’s alpha = 0.85) [22].

### Statistical analysis

Statistical analyses were conducted using Statistical Package for the Social Sciences version 25.0 (SPSS Inc., Chicago, IL, USA). The confirmatory factor analysis was performed using IBM^®^ SPSS^®^ Amos™20.0. The level of significance was defined as p < 0.05. Categorical variables were represented as counts and percentage values. Normal distribution was tested for quantitative variables. Continuous variables with normal or skewed distribution were presented as mean (standard deviation) or median (interquartile range). Group differences were investigated using the independent t-test for normally distributed data, the Mann–Whitney test for skewed data, and the *x*^2^ tests used for categorical variables. Correlation analyses were used to explore relationships between DEPS-R and other constructs hypothesized to covary with DEPS-R scores such as EDE-Q score, diabetes duration, and HbA1c. In line with Cohen, correlations of 0.10–0.29 were interpreted as small, 0.30–0.49 as a medium, and 0.50–1.0 as large [23]. The factorial structure of the Turkish version of DEPS-R for adults was examined by confirmatory factor analysis, and the internal consistency was tested using Cronbach’s alpha coefficient. In previous studies, DEPS-R three-factor model described [maladaptive eating habits, preoccupation with thinness or weight, and maintaining high blood glucose values to lose weight) was tested using confirmatory factor analysis [18–20]. Fit indexes indicate a good fit when: *x*^2^/df ≤ 3 good, ≤ 5 sometimes permissible; Comparative Fit Index (CFI) > 0.95 good fit, > 0.90 traditional fit, > 0.8 sometimes permissible; Root Mean Square Error of Approximation (RMSEA) < 0.05 good, 0.05–0.10 moderate, > 0.10 bad; PCLOSE ≥ 0.05; Normed Fit Index (NFI) ≥ 0.90 good fit; Parsimony Normed Fit Index (pNFI) ≥ 0.50; Turker Lewis Index (TLI) ≥ 0.90 good fit, and Incremental Fit Index (IFI)] ≥ 0.90 good fit.

## Results

The mean age of 100 participants with T1D was 26.4 ± 7.2 years (63% female), the duration of diabetes was 13.1 ± 6.9 years, and diabetes onset age was 13.3 ± 7.5 years. Median (IQ) HbA1c level and BMI value were 8.3 (1.9)% [67.2 (12.9) mmol/mol] and 24.1 (4.8), respectively. There were no significant differences in age, diabetes onset age, HbA1c levels, and BMI values between females and males (Table 1). Seventy-two percent of the participants were on multiple daily injections (MDI) (≥ 4 daily injections), while 28% were on insulin pump therapy. When categorized by BMI, 2% of the participants were underweight, 59% were normal, 34% were overweight, and 5% were obese. The prevalence of participants with a DEPS-R score ≥ 20 was 31%. The Cronbach’s alpha coefficient of the DEPS-R Turkish version was 0.77, suggesting good internal consistency. When assessed by gender, Cronbach’s alpha was 0.82 for females and 0.71 for males. When internal consistency was explored for three factors identified in the confirmatory factor analysis yielding Cronbach's alpha of 0.78 for factor 1 (maladaptive eating habits), 0.64 for factor 2 (preoccupation with thinness), and 0.37 for factor 3 (maintaining high blood glucose levels to lose weight). The Cronbach’s alpha coefficient of the EDE-Q global score for the total sample, females, and males, was 0.91, 0.91, and 0.87, respectively.

**Table 1:** Characteristics of participants

	All (n = 100)	Females (n = 63)	Males (n = 37)	p
Age (years)*	26.4 ± 7.2	26.1 ± 7.1	27.0 ± 7.4	0.504^a^
Diabetes duration (years)*	13.1 ± 6.9	11.9 ± 6.1	15.2 ± 7.8	0.021^a^
Onset age of T1D (years)*	13.3 ± 7.5	14.3 ± 7.9	11.8 ± 6.4	0.139^a^
HbA1c (%) [mmol/mol]**	8.0 (1.9) [64 (21)]	8.1 (2.1) [65 (23)]	7.9 (1.5) [63 (16)]	0.330^b^
BMI (kg/m^2^)**	24.1 (4.8)	23.4 (4.8)	25.1 (5.3)	0.062^b^
Insulin pump therapy, (n; %)	28 (28%)	16 (32.4%)	12 (25.4%)	0.449^c^
Multiple daily injections ≥ 4, (n; %)	72 (72%)	47 (79.4%)	25 (81.1%)	

*Data presented mean ± SD, **Data are median (IR), BMI: Body Mass Index, ^a^Independent sample t test, ^b^Mann Whitney U test, ^c^Chi-square test. NS: no significant. p values refer to the significance of the difference between genders

During the Confirmatory Factor Analysis (CFA) modeling process, we evaluated both 2-factor (*X*^2^/df = 1.831; CFI = 0.882; TLI = 0.835; RMSEA = 0.087) and 3-factor models. However, since the objective of this study was not to compare the performance of various models, only the outcomes of the model demonstrating the best fit are showcased. We began by examining the standard CFA models, where, as a default procedure, we set all error covariances to zero. In the case of the three-factor CFA model, we assigned indicators to load exclusively onto the factors that were initially theorized to represent the constructs they were meant to measure. This means that no indicators were allowed to load onto factors other than the ones they were originally associated with. Model fit was assessed, and specific adjustments were applied to each model, guided by the modification indices (MIs) provided in the computer program’s output. In the best model we have designed for these 16 variables, the structure includes three latent variables (or factors), each of which is represented by three indicators:

- F1 measured by 3 variables: D2, D3, D4, D5, D7, D12, D13, D14 and D15
- F2 measured by 3 variables: D1, D6, D11 and D16
- F3 measured by 3 variables: D8, D9, D10

**Fig. 1:**
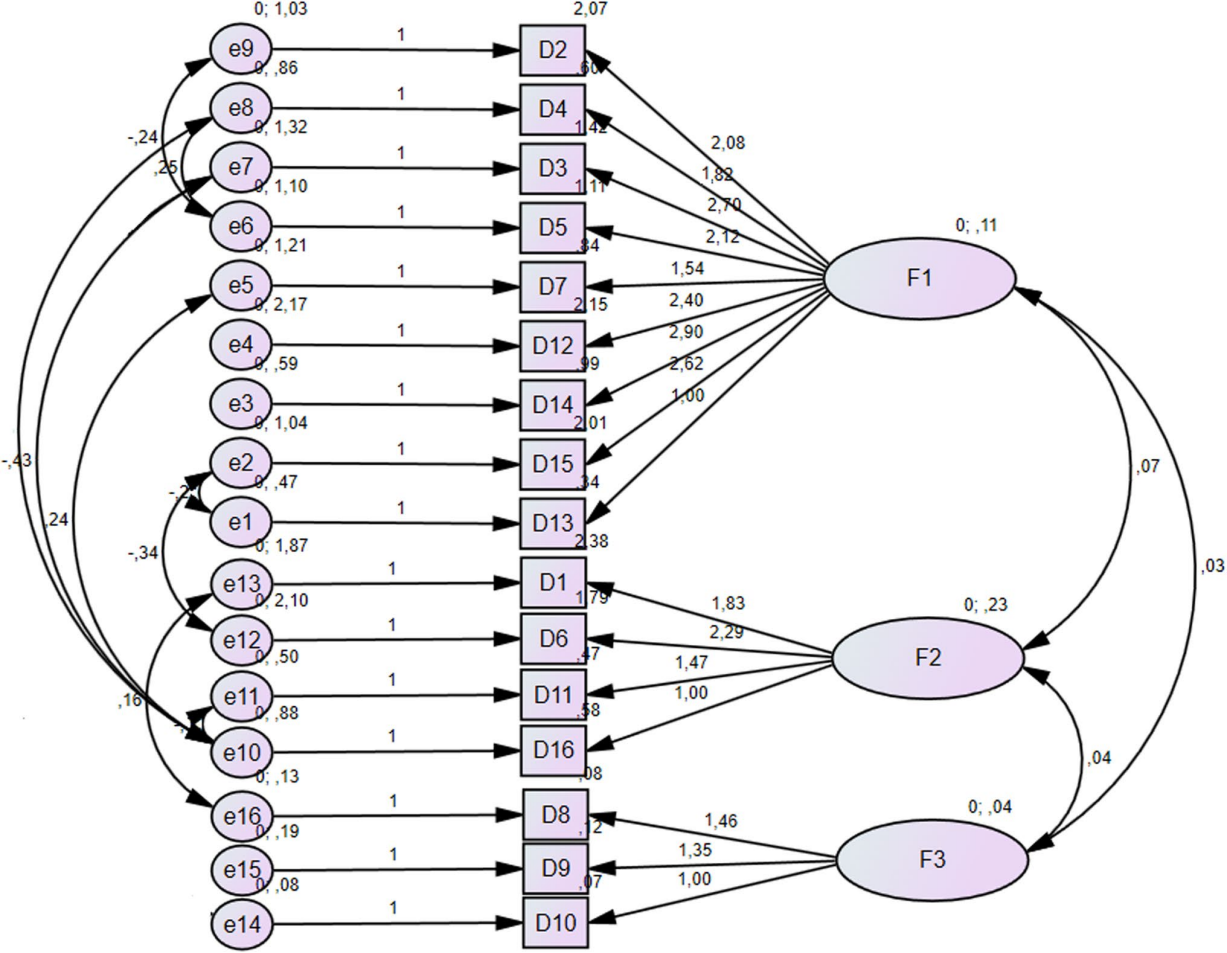
Confirmatory Factor Analysis of the Adults with T1D for DEPS-R

The path diagram of the factorial solution in Fig. 1 and the confirmatory factor analysis results in Table 2 are shown. The confirmatory factor analysis results show that the three-factor model was a good fit.

**Table 2:** Results of confirmatory factor analysis

Goodness of fit index	Acceptable level	Model-fit
*X*^2^/df (CMIN/df)	≤ 3 good; ≤ 5 sometimes permissible	**1.386**
CFI	≥ 0.95 great; ≥ 0.90 traditional; ≥ 0.80 sometimes permissible	**0.901**
RMSEA	< 0.05 good; 0.05–0.10 moderate; > 0.10 bad	**0.062**
PCLOSE	≥ 0.05	**0.217**
NFI	≥ 0.90 good fit	0.816
pNFI	≥ 0.500	**0.544**
TLI	≥ 0.90 good fit	0.878
IFI	≥ 0.90 good fit	**0.903**

Bold charecters represented good fit

*CFI* comperative fit ındex, *RMSEA* root mean square error of approximation, *NFI* normed fit ındex, *pNFI* parsimony normed fit ındex, *TLI* Turker Lewis Index, *IFI* ıncremental fit ındex

The median scores obtained with the DEPS-R for the total sample, females, and males, were 15.0 (13.0), 15.0 (15.0), and 15.0 (11.0), respectively, and was no statistically significant difference between females and males (p > 0.05). The median DEPS-R scores were similar for the MDI group and insulin pump group 15.5 (11.0), and 15.0 (15.0), respectively. A recommended DEPS-R cut-off score of ≥ 20 has been empirically established as a threshold indicating the need for further clinical assessment of eating pathology [10]. A total of 31.7% of the females and 29.7% of the males DEPS-R scores were above this cut-off value, and there was no significant difference between gender (p > 0.05). Sixty-one percent of the participants for whom DEPS-R scored ≥ 20 stated that not receiving enough insulin to cover the food when they overate (Q4), and 42% skipped the following insulin dose after overeating (Q13). While the majority of participants with DEPS-R ≥ 20 were females (64.5%), the proportion of males who indicated that they had insulin restriction was higher (among females 55%, males 73%). The DEPS-R score was significantly large correlated with factor-1 (r = 0.88, p < 0.01), factor-2 (r = 0.67, p < 0.01) and medium correlated with factor 3 (r = 0.36, p < 0.01). While the correlations among females were large-sized for factor-1 and factor-2, the correlation of factor-3 was large-sized among males (Table 3).

**Table 3:** Correlations between the three DEPS-R subfactors and, age, onset age of T1D, diabetes duration, HbA1c, and BMI

	Factor 1	Factor 2	Factor 3
Age (years)	− 0.01	0.15	0.05
Diabetes duration (years)	− 0.03	− 0.09	0.04
Onset age of T1D (years)	− 0.10	0.19	0.07
HbA_1_c (%, mmol/mol)	0.12	0.12	0.09
BMI (kg/m^2^)	0.08	0.35^b^	0.16
DEPS-R total score	0.88^b^	0.67^b^	0.36^b^
EDE-Q global score	0.35^b^	0.75^b^	0.12

Spearman correlation, ^a^differences were significant at p < 0.05, ^b^differences were significant p < 0.01

BMI: Body mass index

The DEPS-R ≥ 20 group had statistically significantly higher EDE-Q global score and BMI than DEPS-R < 20 group (p < 0.001, p < 0.05, respectively). However, diabetes duration, diabetes onset age, and HbAa1c levels were not statistically different between the groups. The median scores obtained with the EDE-Q for the total sample, for females, and males were 0.91 (1.47), 0.98 (1.67), and 0.81 (1.43), respectively and there were female’s EDE-Q scores statistically significantly higher than male’s (p < 0.05).

While the DEPS-R score was significantly large-sized correlated with the EDE-Q global score among females (r = 0.71, p < 0.01), there was a medium-sized correlation among males (r = 0.33, p < 0.05). There was no correlation between the HbA1c values of participants and neither EDE-Q nor DEPS-R scores. However, when split by gender, HbA1c was significantly small-sized correlated with EDE-Q global score (r = 0.27, p < 0.05) and large-sized correlated with DEPS-R score (r = 0.71, p < 0.01) among females.

## Discussion

While there are many studies on the validation of DEPS-R in adolescents with T1D [11–13, 18, 19], the number of studies on the validation of this questionnaire in adults with T1D is limited [14, 16, 24, 25]. Consistent with the previous studies, this study reported acceptable internal consistency and construct validity of the Turkish version of DEPS-R in adults with T1D.

In our previous study, we first used the Turkish version of DEPS-R, which showed that this screening tool had good internal consistency (Cronbach’s alpha = 0.85) in a representative sample of adolescents with T1D [13]. Both the first adult study with the Norwegian version (Cronbach’s alpha = 0.84) and the other adult studies with Chinese (Cronbach’s alpha = 0.78), Spanish (Cronbach’s alpha = 0.82), and Greek (Cronbach’s alpha = 0.89) versions also showed that DEPS-R has a good internal consistency in adults with T1D [14, 16, 24, 25]. Consistent with the previous validation studies, the Turkish version of DEPS-R showed good internal consistency in adults with T1D with a Cronbach’s alpha of 0.77.

In whole sample, unlike in previous studies, males’ median DEPS-R scores in this sample were similar to females but higher than in previous studies males [14, 19, 24]. Unlike other adult studies with DEPS-R, this study was conducted 1 year after the onset of the pandemic. The reason why the DEPS-R score of males in our study group was higher than other adult studies might be due to the unique environmental and psychological factors experienced during the quarantine period during the COVID 19 pandemic which might have caused increased body dissatisfaction and changes in nutrition-exercise related behavior in males. In this sample, where we used DEPS-R as a screening tool, the prevalence of DEPS-R ≥ 20 was 31%, consistent with the other adult studies [16, 25, 26].

Similarly previous adult studies, in our sample participants with DEPS-R scores ≥ 20 had significantly higher BMI levels than with DEPS-R scores < 20 [14, 25, 26]. Considering the social pressure about thinness and/or to be fit, in both genders, individuals with T1D are particularly at risk of weight loss practices such as insulin restriction or omission. Most studies focus on females, but some researchers suggest that males with T1D also may have an increased risk of development of DEB [28]. Similarly, with our previous adolescent study, in our sample, as stated above, males appeared to be at risk for insulin restriction [13]. Weight issues and external appearance may be the main problems in females, and muscularity-oriented dissatisfaction may be a problem in males. Rather than being preoccupied with thinness, males are more likely to be preoccupied with body composition (i.e. fat to muscle ratio) [28]. A diabetes-specific screening tool such as DEPS-R may be essential for detecting the risk in both genders with T1D. However, studies with psychiatric evaluation focusing on males with T1D are needed to better understand the causes of insulin omission and restriction in males. There was no relationship between HbA1c and DEPS-R score in the whole study group (p > 0.05). However, while there was a large correlation between DEPS-R score and HbA1c in females with T1D (p < 0.001) and no relationship between DEPS-R score and HbA1c among males who reported a higher insulin restriction/omission rate than females. The relationship between HbA1c and DEPS-R score differing by genders is similar to the previous adult study [14].When assessing glycemic control for patients prone to glycemic variability, especially people with type 1 diabetes, glycemic control HbA1c may not provide a measure of glycemic variability or hypoglycemia [29]. This may be explained by the fact that in T1D patients with insulin omission/restriction, overtreatment to correct the hyperglycemic state may have contributed to the reduction of HbA1c levels by causing episodes of hypoglycemia. In future studies, it would be helpful to use the data of continuous glucose monitoring systems and HbA1c when assessing metabolic control.

This is the first study to use both confirmatory factor analysis and EDE-Q on the results of the Turkish version of DEPS-R to investigate construct validity. In parallel with the Norwegian study results, there were significant and large correlations with DEPS-R and EDE-Q scores, suggesting good construct validity in adults with T1D [18]. Furthermore, confirmatory factor analysis gave force to the previously reported three-factor solution identifying ‘maladaptive eating’, ‘preoccupation with thinness’, and ‘maintain high blood glucose levels to lose weight’ [13, 14, 16, 18, 19]. Confirmatory factor analysis is a data reduction method used to identify a smaller number of principal components in the observed items [30]. Therefore, the single-factor structure proposed in the original version by Markowitz et al. and the Greek version of DEPS-R by Karastogiannidou et al. does not include detailed definitions such as skipping meals, binge eating, purging, insulin omission, etc., which constitute the risk of DEB [11, 25]. When constructing CFA models, it is crucial to take into account models found in the existing literature. Furthermore, a comprehensive evaluation of the data structure is essential, and enhancements to the models (One or more factorial…) should be implemented through the application of corrections recommended by computer software. Although a single-factor structure is recommended as a screening tool, the multifactor structure is important in providing information about the behaviors that predispose to eating disorders. Finally, it can allow the creation of individual treatment plans.

To further evaluate the reported three-factor solutions the factors were correlated with the DEPS-R total score and EDE-Q total score. All three factors correlated with the DEPS-R total score, furthermore, factor 1 and 2 correlated EDE-Q total score, but no correlation with factor 3. Considering that factor 3 reflects behaviors maintaining high blood sugar levels to lose weight, it was usual that there was no relationship between EDE-Q (which was developed for use in individuals without T1D), and factor 3. In previous studies, factor 1 was more strongly correlated with the DEPS-R total score than factors 2 and 3 suggesting factor 1 to be most dominant factor in the reported three-factor structure [13, 14, 18].

### Strength and limits

The current study is the first study among adults to validate the Turkish version of DEPS-R against the EDE-Q, the preferred measure of specific eating disorder psychopathology. However, the study is limited by its cross-sectional design. A major limitation of this study is its inability to validate the Turkish version of DEPS-R with a structured clinical diagnostic interview by a psychiatrist. Further limitations were that this study was cross-sectional and limited to a single tertiary care diabetes center, which may limit generalisability.

### What is already known on this topic?

- Early detection and treatment of eating disorders/disordered eating behaviors in individuals with T1D are necessary because of potentially severe consequences.
- It is essential to use a screening measure designed specifically for individuals with T1D when assessing disturbed eating behaviors in this population.

### What does this study adds?

- So far, no validated disease-specific short-screening tool for adults with T1D in Turkey has been established.
- The Turkish version of DEPS-R is a valid screening tool for disordered eating behaviors in T1D.
- A short, self-administered diabetes-specific screening tool for disordered eating behavior can be used routinely in the clinical care of adults with T1D.

## Conclusion

Early detection and treatment of eating disorders in individuals with T1D are necessary because of these potentially severe consequences. Therefore, it is essential to use a screening tool designed specifically for individuals with T1D when assessing DEB in this population. So far, no validated disease-specific short screening tool for adults with T1D in Turkey has been established. The Turkish version of DEPS-R is a valid screening tool for DEB in adults with T1D. However, confirmatory factor analysis supports three-factor solutions describing ‘maladaptive eating’, ‘preoccupation with thinness’, and ‘maintaining high blood glucose levels to lose weight’. A short, self-administered diabetes-specific screening tool for DEB can be used routinely in the clinical care of adults with T1D. Future studies should focus on the validity of the DEPS-R by comparing it to a structured clinical diagnostic interview conducted by a professional.

## Data Availability

Not applicable.
